# Supplementary material for: A homogeneous split-luciferase assay for rapid and sensitive detection of anti-SARS CoV-2 antibodies
Source: Nat Commun. 2021 Mar 22;12:1806. doi: 10.1038/s41467-021-22102-6 (PMC7985487; doi:10.1038/s41467-021-22102-6)
Supplement: Supplementary file 1 — Supplementary Information [file 41467_2021_22102_MOESM1_ESM.pdf]

Supplementary methods: nucleotide sequences of the constructs

1. Δ11S

```
  1 ATGggccatc atcatcatca tcatcatcat ATGgttttta cacttgaaga ttttgtgggt
 61 gattgggaac aaactgctgc atataattta gatcaagttt tagaacaggg tggagtgagt
121 tctcttttac aaaatcttgc agtctcagtt acaccaatac aaagaatagt tagaagtgga
181 gaaaatgcat taaagatcga tatacatgta ataattcctt atgagggact atcagcagac
241 caaatggcac aaattgagga agtattcaaa gttgtatatc cagtagacga tcatcacttt
301 aaagtaatat taccttatgg aactttagta attgatggtg taacaccaaa tatgttaaat
361 tattttggta gaccatacga agggattgca gtttttgatg gaaagaaaat aaccgtaaca
421 ggcactttgt ggaatggaaa taaaataata gatgaaaggt taattacacc tgatTAA
```

2. β9-G

```
  1 ATGGGCTCCA TGCTGTTCCG AGTAACCATC AACAGTggag gtggatccGG TGGTGGAGGG
 61 AGCacctaca aacttGtcAT Taacggtaaa accctgaaag gtgaaaccac caccgaagct
121 gttgacgctg ctaccgcgga aaaagttttc aaacagtacg ctaacgacaa cggtgttgac
181 ggtgaatgga cctacgacga cgctaccaaa acctTcaccg taacggaaGG TGGCGGTAGC
241 catcaccacc atcaccacTG A
```

3. G-β9

```
  1 ATGggccatc atcatcatca tcatGGTGGA GGGAGCacct acaaacttGt cATTaacggt
 61 aaaaccctga aaggtgaaac caccaccgaa gctgttgacg ctgctaccgc ggaaaaagtt
121 ttcaaacagt acgctaacga caacggtgtt gacggtgaat ggacctacga cgacgctacc
181 aaaacctTca ccgtaacgga aGGTGGCGGT AGCggtggcg gaggctctGG CTCCATGCTG
241 TTCCGAGTAA CCATCAACAG Ttaa
```

4. β9-G-β9

```
  1 ATGGGCTCCA TGCTGTTCCG AGTAACCATC AACAGTggag gtggatccGG TGGTGGAGGG
 61 AGCacctaca aacttGtcAT Taacggtaaa accctgaaag gtgaaaccac caccgaagct
121 gttgacgctg ctaccgcgga aaaagttttc aaacagtacg ctaacgacaa cggtgttgac
181 ggtgaatgga cctacgacga cgctaccaaa acctTcaccg taacggaaGG AGGAGGATCT
241 GGAGGTGGAT CCGGCTCCAT GCTGTTCCGA GTAACCATCA ACAGTGGCGG AAGCCATCAT
301 CACCACCATC ATCACCATTG A
```

5. β10-RBD

```
  1 ATGCGGCTTC CGGGTGCGAT GCCAGCTCTG GCCCTCAAAG GCGAGCTGCT GTTGCTGTCT
 61 CTCCTGTTAC TTCTGGAACC ACAGATCTCT CAGGGCGGTG GaGTGAGCGG CTGGCGGCTG
121 TTCAAGAAGA TTAGCGGTGG CGGAGGATCT GGAGGTGGAT CCagggtgca gccaaccgag
181 tctatcgtgc gctttcctaa tatcacaaac ctgtgcccat ttggcgaggt gttcaacgca
241 acccgcttcg ccagcgtgta cgcctggaat aggaagcgga tcagcaactg cgtggccgac
301 tatagcgtgc tgtacaactc cgcctctttc agcaccttta agtgctatgg cgtgtccccc
361 acaaagctga atgacctgtg ctttaccaac gtctacgccg attctttcgt gatcaggggc
421 gacgaggtgc gccagatcgc ccccggccag acaggcaaga tcgcagacta caattataag
481 ctgccagacg atttcaccgg ctgcgtgatc gcctggaaca gcaacaatct ggattccaaa
541 gtgggcggca actacaatta tctgtaccgg ctgtttagaa agagcaatct gaagcccttc
601 gagagggaca tctctacaga aatctaccag gccggcagca ccccttgcaa tggcgtggag
661 ggctttaact gttatttccc actccagtcc tacggcttcc agcccacaaa cggcgtgggc
721 tatcagcctt accgcgtggt ggtgctgagc tttgagctgc tgcacgcccc agcaacagtg
781 tgcggcccca agaagtccac caatctggtg aagaacaagt gcgtgaactt cGGCGGAAGC
841 CATCATCACC ACCATCATCA CCATTGA
```

6. RBD-β10

```
  1 atgttcgtct tcctggtcct gctgcctctg gtctcctcac agagggtgca gccaaccgag
 61 tctatcgtgc gctttcctaa tatcacaaac ctgtgcccat ttggcgaggt gttcaacgca
121 acccgcttcg ccagcgtgta cgcctggaat aggaagcgga tcagcaactg cgtggccgac
181 tatagcgtgc tgtacaactc cgcctctttc agcaccttta agtgctatgg cgtgtccccc
241 acaaagctga atgacctgtg ctttaccaac gtctacgccg attctttcgt gatcaggggc
```

```
 301 gacgaggtgc gccagatcgc ccccggccag acaggcaaga tcgcagacta caattataag
 361 ctgccagacg atttcaccgg ctgcgtgatc gcctggaaca gcaacaatct ggattccaaa
 421 gtgggcggca actacaatta tctgtaccgg ctgtttagaa agagcaatct gaagcccttc
 481 gagagggaca tctctacaga aatctaccag gccggcagca ccccttgcaa tggcgtggag
 541 ggctttaact gttatttccc actccagtcc tacggcttcc agcccacaaa cggcgtgggc
 601 tatcagcctt accgcgtggt ggtgctgagc tttgagctgc tgcacgcccc agcaacagtg
 661 tgcggcccca agaagtccac caatctggtg aagaacaagt gcgtgaactt cGGCGGAGGA
 721 GGATCTGGAG GTGGATCCGT GAGCGGCTGG CGGCTGTTCA AGAAGATTAG CGGCGGAAGC
 781 CATCATCACC ACCATCATCA CCATTGA
```

7. β10-RBD-β10

```
   1 ATGCGGCTTC CGGGTGCGAT GCCAGCTCTG GCCCTCAAAG GCGAGCTGCT GTTGCTGTCT
  61 CTCCTGTTAC TTCTGGAACC ACAGATCTCT CAGGGCGGTG GaGTGAGCGG CTGGCGGCTG
 121 TTCAAGAAGA TTAGCGGTGG CGGAGGATCT GGAGGTGGAT CCagggtgca gccaaccgag
 181 tctatcgtgc gctttcctaa tatcacaaac ctgtgcccat ttggcgaggt gttcaacgca
 241 acccgcttcg ccagcgtgta cgcctggaat aggaagcgga tcagcaactg cgtggccgac
 301 tatagcgtgc tgtacaactc cgcctctttc agcaccttta agtgctatgg cgtgtccccc
 361 acaaagctga atgacctgtg ctttaccaac gtctacgccg attctttcgt gatcaggggc
 421 gacgaggtgc gccagatcgc ccccggccag acaggcaaga tcgcagacta caattataag
 481 ctgccagacg atttcaccgg ctgcgtgatc gcctggaaca gcaacaatct ggattccaaa
 541 gtgggcggca actacaatta tctgtaccgg ctgtttagaa agagcaatct gaagcccttc
 601 gagagggaca tctctacaga aatctaccag gccggcagca ccccttgcaa tggcgtggag
 661 ggctttaact gttatttccc actccagtcc tacggcttcc agcccacaaa cggcgtgggc
 721 tatcagcctt accgcgtggt ggtgctgagc tttgagctgc tgcacgcccc agcaacagtg
 781 tgcggcccca agaagtccac caatctggtg aagaacaagt gcgtgaactt cGGCGGAGGA
 841 GGATCTGGAG GTGGATCCGT GAGCGGCTGG CGGCTGTTCA AGAAGATTAG CGGCGGAAGC
 901 CATCATCACC ACCATCATCA CCATTGA
```

8. β10-S

```
   1 ATGCGGCTTC CGGGTGCGAT GCCAGCTCTG GCCCTCAAAG GCGAGCTGCT GTTGCTGTCT
  61 CTCCTGTTAC TTCTGGAACC ACAGATCTCT CAGGGCGGTG GaGTGAGCGG CTGGCGGCTG
 121 TTCAAGAAGA TTAGCGGTGG CGGAGGATCT GGAGGTGGAT CCtgcgtcaa tctgacaact
 181 cggactcagc tgccacctgc ttatactaat agcttcacca gaggcgtgta ctatcctgac
 241 aaggtgttta gaagctccgt gctgcactct acacaggatc tgtttctgcc attctttagc
 301 aacgtgacct ggttccacgc catccacgtg agcggcacca atggcacaaa gcggttcgac
 361 aatcccgtgc tgccttttaa cgatggcgtg tacttcgcct ctaccgagaa gagcaacatc
 421 atcagaggct ggatctttgg caccacactg gactccaaga cacagtctct gctgatcgtg
 481 aacaatgcca ccaacgtggt catcaaggtg tgcgagttcc agttttgtaa tgatcccttc
 541 ctgggcgtgt actatcacaa gaacaataag agctggatgg agtccgagtt tagagtgtat
 601 tctagcgcca acaactgcac atttgagtac gtgagccagc ctttcctgat ggacctggag
 661 ggcaagcagg gcaatttcaa gaacctgagg gagttcgtgt ttaagaatat cgacggctac
 721 ttcaaaatct actctaagca cacccccatc aacctggtgc gcgacctgcc tcagggcttc
 781 agcgccctgg agcccctggt ggatctgcct atcggcatca acatcacccg gtttcagaca
 841 ctgctggccc tgcacagaag ctacctgaca cccggcgact cctctagcgg atggaccgcc
 901 ggcgctgccg cctactatgt gggctacctc cagccccgga ccttcctgct gaagtacaac
 961 gagaatggca ccatcacaga cgcagtggat tgcgccctgg acccccctgag cgagacaaag
1021 tgtacactga agtcctttac cgtggagaag ggcatctatc agacatccaa tttcagggtg
1081 cagccaaccg agtctatcgt gcgctttcct aatatcacaa acctgtgccc atttggcgag
1141 gtgttcaacg caacccgctt cgccagcgtg tacgcctgga ataggaagcg gatcagcaac
1201 tgcgtggccg actatagcgt gctgtacaac tccgcctctt tcagcacctt taagtgctat
1261 ggcgtgtccc ccacaaagct gaatgacctg tgctttacca acgtctacgc cgattctttc
1321 gtgatcaggg gcgacgaggt gcgccagatc gcccccggcc agacaggcaa gatcgcagac
1381 tacaattata agctgccaga cgatttcacc ggctgcgtga tcgcctggaa cagcaacaat
1441 ctggattcca aagtgggcgg caactacaat tatctgtacc ggctgtttag aaagagcaat
1501 ctgaagccct tcgagaggga catctctaca gaaatctacc aggccggcag caccccttgc
1561 aatggcgtgg agggctttaa ctgttatttc ccactccagt cctacggctt ccagcccaca
1621 aacggcgtgg gctatcagcc ttaccgcgtg gtggtgctga gctttgagct gctgcacgcc
```

```
1681 ccagcaacag tgtgcggccc caagaagtcc accaatctgg tgaagaacaa gtgcgtgaac
1741 ttcaacttca acggcctgac cggcacaggc gtgctgaccg agtccaacaa gaagttcctg
1801 ccatttcagc agttcggcag ggacatcgca gataccacag acgccgtgcg cgacccacag
1861 accctggaga tcctggacat cacaccctgc tctttcggcg gcgtgagcgt gatcacaccc
1921 ggcaccaata caagcaacca ggtggccgtg ctgtatcagg acgtgaattg taccgaggtg
1981 cccgtggcta tccacgccga tcagctgacc ccaacatggc gggtgtacag caccggctcc
2041 aacgtcttcc agacaagagc cggatgcctg atcggagcag agcacgtgaa caattcctat
2101 gagtgcgaca tcccaatcgg cgccggcatc tgtgcctctt accagaccca gacaaactct
2161 cccGgaagCg ccAgCagcgt ggcctcccag tctatcatcg cctataccat gtccctgggc
2221 gccgagaaca gcgtggccta ctctaacaat agcatcgcca tcccaaccaa cttcacaatc
2281 tctgtgacca cagagatcct gcccgtgtcc atgaccaaga catctgtgga ctgcacaatg
2341 tatatctgtg gcgattctac cgagtgcagc aacctgctgc tccagtacgg cagcttttgt
2401 acccagctga atagagccct gacaggcatc gccgtggagc aggataagaa cacacaggag
2461 gtgttcgccc aggtgaagca aatctacaag acccccccta tcaaggactt tggcggcttc
2521 aatttttccc agatcctgcc tgatccatcc aagccttcta agcggagctt tatcgaggac
2581 ctgctgttca acaaggtgac cctggccgat gccggcttca tcaagcagta tggcgattgc
2641 ctgggcgaca tcgcagccag ggacctgatc tgcgcccaga agtttaatgg cctgaccgtg
2701 ctgccacccc tgctgacaga tgagatgatc gcacagtaca caagcgccct gctggccggc
2761 accatcacat ccggatggac cttcggcgca ggagccgccc tccagatccc ctttgccatg
2821 cagatggcct ataggttcaa cggcatcggc gtgacccaga atgtgctgta cgagaaccag
2881 aagctgatcg ccaatcagtt taactccgcc atcggcaaga tccaggacag cctgtcctct
2941 acagccagcg ccctgggcaa gctccaggat gtggtgaatc agaacgccca ggccctgaat
3001 accctggtga agcagctgag cagcaacttc ggcgccatct ctagcgtgct gaatgacatc
3061 ctgagccggc tggacCCTCC Tgaggcagag gtgcagatcg accggctgat caccggccgg
3121 ctccagagcc tccagaccta tgtgacacag cagctgatca gggccgccga gatcagggcc
3181 agcgccaatc tggcagcaac caagatgtcc gagtgcgtgc tgggccagtc taagagagtg
3241 gacttttgtg gcaagggcta tcacctgatg tccttccctc agtctgcccc acacggcgtg
3301 gtgtttctgc acgtgaccta cgtgcccgcc caggagaaga acttcaccac agcccctgcc
3361 atctgccacg atggcaaggc ccactttcca agggagggcg tgttcgtgtc caacggcacc
3421 cactggtttg tgacacagcg caatttctac gagccccaga tcatcaccac agacaacacc
3481 ttcgtgagcg gcaactgtga cgtggtcatc ggcatcgtga acaataccgt gtatgatcca
3541 ctccagcccg agctggacag ctttaaggag gagctggata agtatttcaa gaatcacacc
3601 tcccctgacg tggatctggg cgacatcagc ggcatcaatg cctccgtggt gaacatccag
3661 aaggagatcg accgcctgaa cgaggtggct aagaatctga acgagagcct gatcgacctc
3721 caggagctgg gcaagtatga gcagtacatc aagtggcccG GaGGCAGCGG aGGCTACATC
3781 CCCGAGGCCC CtCGCGAtGG aCAGGCtTAC GTGCGCAAGG ACGGCGAGTG GGTGCTGCTG
3841 AGCACCTTCC TGGGCGGAAG CCATCATCAC CACCATCATC ACCATTGA
```

9. S-β10

```
   1 atgttcgtct tcctggtcct gctgcctctg gtctcctcac agtgcgtcaa tctgacaact
  61 cggactcagc tgccacctgc ttatactaat agcttcacca gaggcgtgta ctatcctgac
 121 aaggtgttta gaagctccgt gctgcactct acacaggatc tgtttctgcc attctttagc
 181 aacgtgacct ggttccacgc catccacgtg agcggcacca atggcacaaa gcggttcgac
 241 aatcccgtgc tgccttttaa cgatggcgtg tacttcgcct ctaccgagaa gagcaacatc
 301 atcagaggct ggatctttgg caccacactg gactccaaga cacagtctct gctgatcgtg
 361 aacaatgcca ccaacgtggt catcaaggtg tgcgagttcc agttttgtaa tgatcccttc
 421 ctgggcgtgt actatcacaa gaacaataag agctggatgg agtccgagtt tagagtgtat
 481 tctagcgcca acaactgcac atttgagtac gtgagccagc ctttcctgat ggacctggag
 541 ggcaagcagg gcaatttcaa gaacctgagg gagttcgtgt ttaagaatat cgacggctac
 601 ttcaaaatct actctaagca cacccccatc aacctggtgc gcgacctgcc tcagggcttc
 661 agcgccctgg agcccctggt ggatctgcct atcggcatca acatcacccg gtttcagaca
 721 ctgctggccc tgcacagaag ctacctgaca cccggcgact cctctagcgg atggaccgcc
 781 ggcgctgccg cctactatgt gggctacctc cagccccgga ccttcctgct gaagtacaac
 841 gagaatggca ccatcacaga cgcagtggat tgcgccctgg accccctgag cgagacaaag
 901 tgtacactga agtcctttac cgtggagaag ggcatctatc agacatccaa tttcagggtg
 961 cagccaaccg agtctatcgt gcgctttcct aatatcacaa acctgtgccc atttggcgag
1021 gtgttcaacg caacccgctt cgccagcgtg tacgcctgga ataggaagcg gatcagcaac
```

1081 tgcgtggccg actatagcgt gctgtacaac tccgcctctt tcagcacctt taagtgctat
1141 ggcgtgtccc ccacaaagct gaatgacctg tgctttacca acgtctacgc cgattctttc
1201 gtgatcaggg gcgacgaggt gcgccagatc gcccccggcc agacaggcaa gatcgcagac
1261 tacaattata agctgccaga cgatttcacc ggctgcgtga tcgcctggaa cagcaacaat
1321 ctggattcca aagtgggcgg caactacaat tatctgtacc ggctgtttag aaagagcaat
1381 ctgaagccct tcgagaggga catctctaca gaaatctacc aggccggcag cacccccttgc
1441 aatggcgtgg agggctttaa ctgttatttc ccactccagt cctacggctt ccagcccaca
1501 aacggcgtgg gctatcagcc ttaccgcgtg gtggtgctga gctttgagct gctgcacgcc
1561 ccagcaacag tgtgcggccc caagaagtcc accaatctgg tgaagaacaa gtgcgtgaac
1621 ttcaacttca acggcctgac cggcacaggc gtgctgaccg agtccaacaa gaagttcctg
1681 ccatttcagc agttcggcag ggacatcgca gataccacag acgccgtgcg cgacccacag
1741 accctggaga tcctggacat cacaccctgc tctttcggcg gcgtgagcgt gatcacaccc
1801 ggcaccaata caagcaacca ggtggccgtg ctgtatcagg acgtgaattg taccgaggtg
1861 cccgtggcta tccacgccga tcagctgacc ccaacatggc gggtgtacag caccggctcc
1921 aacgtcttcc agacaagagc cggatgcctg atcggagcag agcacgtgaa caattcctat
1981 gagtgcgaca tcccaatcgg cgccggcatc tgtgcctctt accagaccca gacaaactct
2041 cccGgaagCg ccAgCagcgt ggcctcccag tctatcatcg cctataccat gtccctgggc
2101 gccgagaaca gcgtggccta ctctaacaat agcatcgcca tcccaaccaa cttcacaatc
2161 tctgtgacca cagagatcct gcccgtgtcc atgaccaaga catctgtgga ctgcacaatg
2221 tatatctgtg gcgattctac cgagtgcagc aacctgctgc tccagtacgg cagcttttgt
2281 acccagctga atagagccct gacaggcatc gccgtggagc aggataagaa cacacaggag
2341 gtgttcgccc aggtgaagca aatctacaag acccccccta tcaaggactt tggcggcttc
2401 aatttttccc agatcctgcc tgatccatcc aagccttcta agcggagctt tatcgaggac
2461 ctgctgttca acaaggtgac cctggccgat gccggcttca tcaagcagta tggcgattgc
2521 ctgggcgaca tcgcagccag ggacctgatc tgcgcccaga agtttaatgg cctgaccgtg
2581 ctgccacccc tgctgacaga tgagatgatc gcacagtaca caagcgccct gctggccggc
2641 accatcacat ccggatggac cttcggcgca ggagccgccc tccagatccc ctttgccatg
2701 cagatggcct ataggttcaa cggcatcggc gtgacccaga atgtgctgta cgagaaccag
2761 aagctgatcg ccaatcagtt taactccgcc atcggcaaga tccaggacag cctgtcctct
2821 acagccagcg ccctgggcaa gctccaggat gtggtgaatc agaacgccca ggccctgaat
2881 accctggtga agcagctgag cagcaacttc ggcgccatct ctagcgtgct gaatgacatc
2941 ctgagccggc tggacCCTCC Tgaggcagag gtgcagatcg accggctgat caccggccgg
3001 ctccagagcc tccagaccta tgtgacacag cagctgatca gggccgccga gatcagggcc
3061 agcgccaatc tggcagcaac caagatgtcc gagtgcgtgc tgggccagtc taagagagtg
3121 gacttttgtg gcaagggcta tcacctgatg tccttccctc agtctgcccc acacggcgtg
3181 gtgtttctgc acgtgaccta cgtgcccgcc caggagaaga acttcaccac agcccctgcc
3241 atctgccacg atggcaaggc ccactttcca agggagggcg tgttcgtgtc caacggcacc
3301 cactggtttg tgacacagcg caatttctac gagccccaga tcatcaccac agacaacacc
3361 ttcgtgagcg gcaactgtga cgtggtcatc ggcatcgtga acaataccgt gtatgatcca
3421 ctccagcccg agctggacag ctttaaggag gagctggata agtatttcaa gaatcacacc
3481 tcccctgacg tggatctggg cgacatcagc ggcatcaatg cctccgtggt gaacatccag
3541 aaggagatcg accgcctgaa cgaggtggct aagaatctga acgagagcct gatcgacctc
3601 caggagctgg gcaagtatga gcagtacatc aagtggcccG GaGGCAGCGG aGGCTACATC
3661 CCCGAGGCCC CtCGCGAtGG aCAGGCtTAC GTGCGCAAGG ACGGCGAGTG GGTGCTGCTG
3721 AGCACCTTCC TGGGCGGAGG AGGATCTGGA GGTGGATCCG TGAGCGGCTG GCGGCTGTTC
3781 AAGAAGATTA GCGGCGGAAG CCATCATCAC CACCATCATC ACCATTGA

10. β10-S-β10

1 ATGCGGCTTC CGGGTGCGAT GCCAGCTCTG GCCCTCAAAG GCGAGCTGCT GTTGCTGTCT
61 CTCCTGTTAC TTCTGGAACC ACAGATCTCT CAGGGCGGTG GaGTGAGCGG CTGGCGGCTG
121 TTCAAGAAGA TTAGtGGTGG CGGAGGATCT GGAGGTGGAT CCtgcgtcaa tctgacaact
181 cggactcagc tgccacctgc ttatactaat agcttcacca gaggcgtgta ctatcctgac
241 aaggtgttta gaagctccgt gctgcactct acacaggatc tgtttctgcc attctttagc
301 aacgtgacct ggttccacgc catccacgtg agcggcacca atggcacaaa gcggttcgac
361 aatcccgtgc tgccttttaa cgatggcgtg tacttcgcct ctaccgagaa gagcaacatc
421 atcagaggct ggatctttgg caccacactg gactccaaga cacagtctct gctgatcgtg
481 aacaatgcca ccaacgtggt catcaaggtg tgcgagttcc agttttgtaa tgatcccttc

541 ctgggcgtgt actatcacaa gaacaataag agctggatgg agtccgagtt tagagtgtat
601 tctagcgcca acaactgcac atttgagtac gtgagccagc ctttcctgat ggacctggag
661 ggcaagcagg gcaatttcaa gaacctgagg gagttcgtgt ttaagaatat cgacggctac
721 ttcaaaatct actctaagca cacccccatc aacctggtgc gcgacctgcc tcagggcttc
781 agcgccctgg agcccctggt ggatctgcct atcggcatca acatcacccg gtttcagaca
841 ctgctggccc tgcacagaag ctacctgaca cccggcgact cctctagcgg atggaccgcc
901 ggcgctgccg cctactatgt gggctacctc cagccccgga ccttcctgct gaagtacaac
961 gagaatggca ccatcacaga cgcagtggat tgcgccctgg acccccctgag cgagacaaag
1021 tgtacactga agtcctttac cgtggagaag ggcatctatc agacatccaa tttcagggtg
1081 cagccaaccg agtctatcgt gcgctttcct aatatcacaa acctgtgccc atttggcgag
1141 gtgttcaacg caacccgctt cgccagcgtg tacgcctgga ataggaagcg gatcagcaac
1201 tgcgtggccg actatagcgt gctgtacaac tccgcctctt tcagcacctt taagtgctat
1261 ggcgtgtccc ccacaaagct gaatgacctg tgctttacca acgtctacgc cgattctttc
1321 gtgatcaggg gcgacgaggt gcgccagatc gcccccggcc agacaggcaa gatcgcagac
1381 tacaattata agctgccaga cgatttcacc ggctgcgtga tcgcctggaa cagcaacaat
1441 ctggattcca aagtgggcgg caactacaat tatctgtacc ggctgtttag aaagagcaat
1501 ctgaagccct tcgagaggga catctctaca gaaatctacc aggccggcag caccccttgc
1561 aatggcgtgg agggctttaa ctgttatttc ccactccagt cctacggctt ccagcccaca
1621 aacggcgtgg gctatcagcc ttaccgcgtg gtggtgctga gctttgagct gctgcacgcc
1681 ccagcaacag tgtgcggccc caagaagtcc accaatctgg tgaagaacaa gtgcgtgaac
1741 ttcaacttca acggcctgac cggcacaggc gtgctgaccg agtccaacaa gaagttcctg
1801 ccatttcagc agttcggcag ggacatcgca gataccacag acgccgtgcg cgacccacag
1861 accctggaga tcctggacat cacaccctgc tctttcggcg gcgtgagcgt gatcacaccc
1921 ggcaccaata caagcaacca ggtggccgtg ctgtatcagg acgtgaattg taccgaggtg
1981 cccgtggcta tccacgccga tcagctgacc ccaacatggc gggtgtacag caccggctcc
2041 aacgtcttcc agacaagagc cggatgcctg atcggagcag agcacgtgaa caattcctat
2101 gagtgcgaca tcccaatcgg cgccggcatc tgtgcctctt accagaccca gacaaactct
2161 cccGgaagCg ccAgCagcgt ggcctcccag tctatcatcg cctataccat gtccctgggc
2221 gccgagaaca gcgtggccta ctctaacaat agcatcgcca tcccaaccaa cttcacaatc
2281 tctgtgacca cagagatcct gcccgtgtcc atgaccaaga catctgtgga ctgcacaatg
2341 tatatctgtg gcgattctac cgagtgcagc aacctgctgc tccagtacgg cagcttttgt
2401 acccagctga atagagccct gacaggcatc gccgtggagc aggataagaa cacacaggag
2461 gtgttcgccc aggtgaagca aatctacaag acccccccta tcaaggactt tggcggcttc
2521 aatttttccc agatcctgcc tgatccatcc aagccttcta agcggagctt tatcgaggac
2581 ctgctgttca acaaggtgac cctggccgat gccggcttca tcaagcagta tggcgattgc
2641 ctgggcgaca tcgcagccag ggacctgatc tgcgcccaga agtttaatgg cctgaccgtg
2701 ctgccacccc tgctgacaga tgagatgatc gcacagtaca caagcgccct gctggccggc
2761 accatcacat ccggatggac cttcggcgca ggagccgccc tccagatccc ctttgccatg
2821 cagatggcct ataggttcaa cggcatcggc gtgacccaga atgtgctgta cgagaaccag
2881 aagctgatcg ccaatcagtt taactccgcc atcggcaaga tccaggacag cctgtcctct
2941 acagccagcg ccctgggcaa gctccaggat gtggtgaatc agaacgccca ggccctgaat
3001 accctggtga agcagctgag cagcaacttc ggcgccatct ctagcgtgct gaatgacatc
3061 ctgagccggc tggacCCTCC Tgaggcagag gtgcagatcg accggctgat caccggccgg
3121 ctccagagcc tccagaccta tgtgacacag cagctgatca gggccgccga gatcagggcc
3181 agcgccaatc tggcagcaac caagatgtcc gagtgcgtgc tgggccagtc taagagagtg
3241 gacttttgtg gcaagggcta tcacctgatg tccttccctc agtctgcccc acacggcgtg
3301 gtgtttctgc acgtgaccta cgtgcccgcc caggagaaga acttcaccac agcccctgcc
3361 atctgccacg atggcaaggc ccactttcca agggagggcg tgttcgtgtc caacggcacc
3421 cactggtttg tgacacagcg caatttctac gagccccaga tcatcaccac agacaacacc
3481 ttcgtgagcg gcaactgtga cgtggtcatc ggcatcgtga acaataccgt gtatgatcca
3541 ctccagcccg agctggacag ctttaaggag gagctggata agtatttcaa gaatcacacc
3601 tcccctgacg tggatctggg cgacatcagc ggcatcaatg cctccgtggt gaacatccag
3661 aaggagatcg accgcctgaa cgaggtggct aagaatctga acgagagcct gatcgacctc
3721 caggagctgg gcaagtatga gcagtacatc aagtggcccG GaGGCAGCGG aGGCTACATC
3781 CCCGAGGCCC CtCGCGAtGG aCAGGCtTAC GTGCGCAAGG ACGGCGAGTG GGTGCTGCTG
3841 AGCACCTTCC TGGGCGGAGG AGGATCTGGA GGTGGATCCG TGAGCGGCTG GCGGCTGTTC
3901 AAGAAGATTA GCGGCGGAAG CCATCATCAC CACCATCATC ACCATTGA

Supplementary Fig. 1

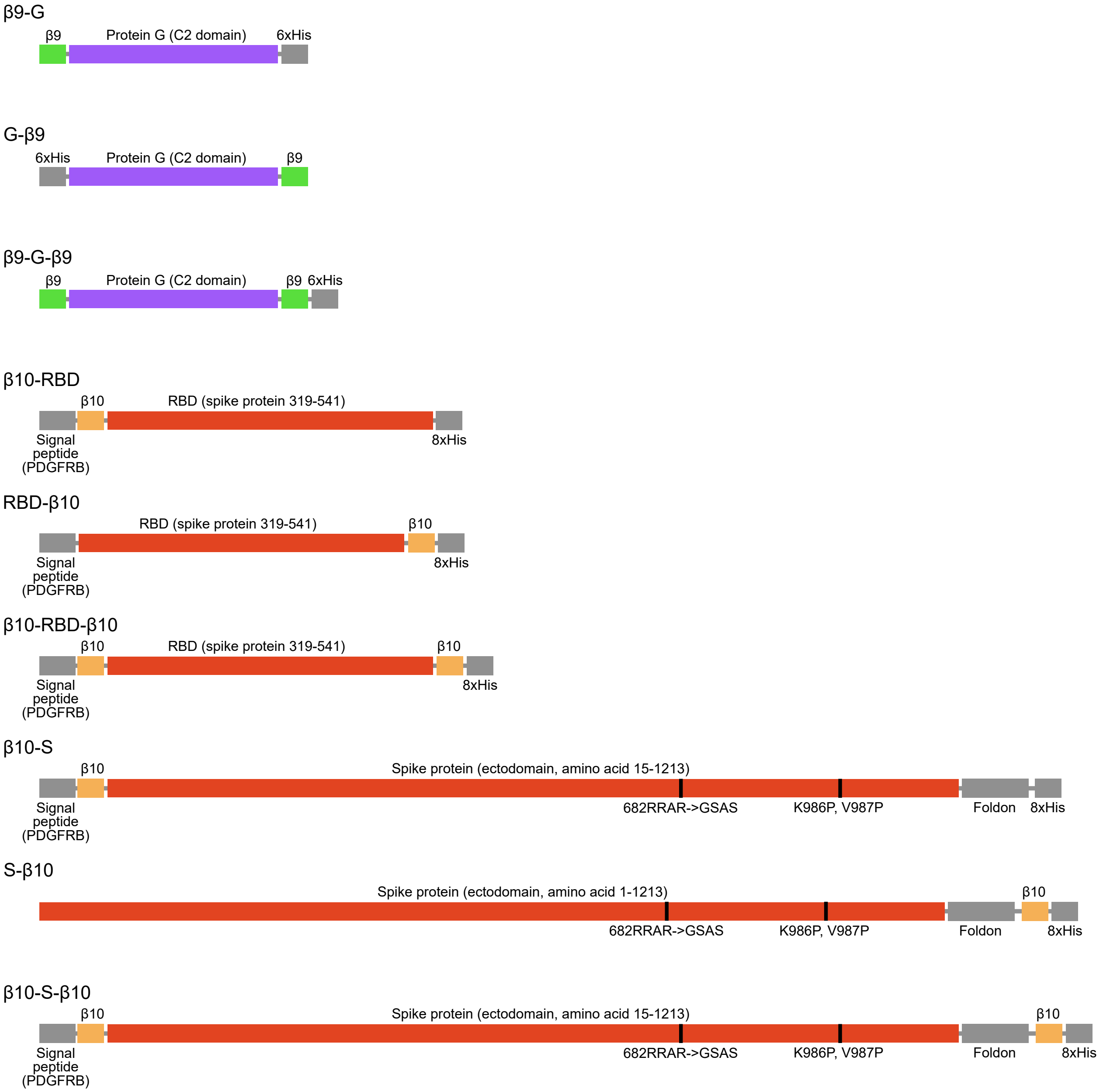

Supplementary Figure 1. Schematic representation of SATiN probes.

Supplementary Fig. 2

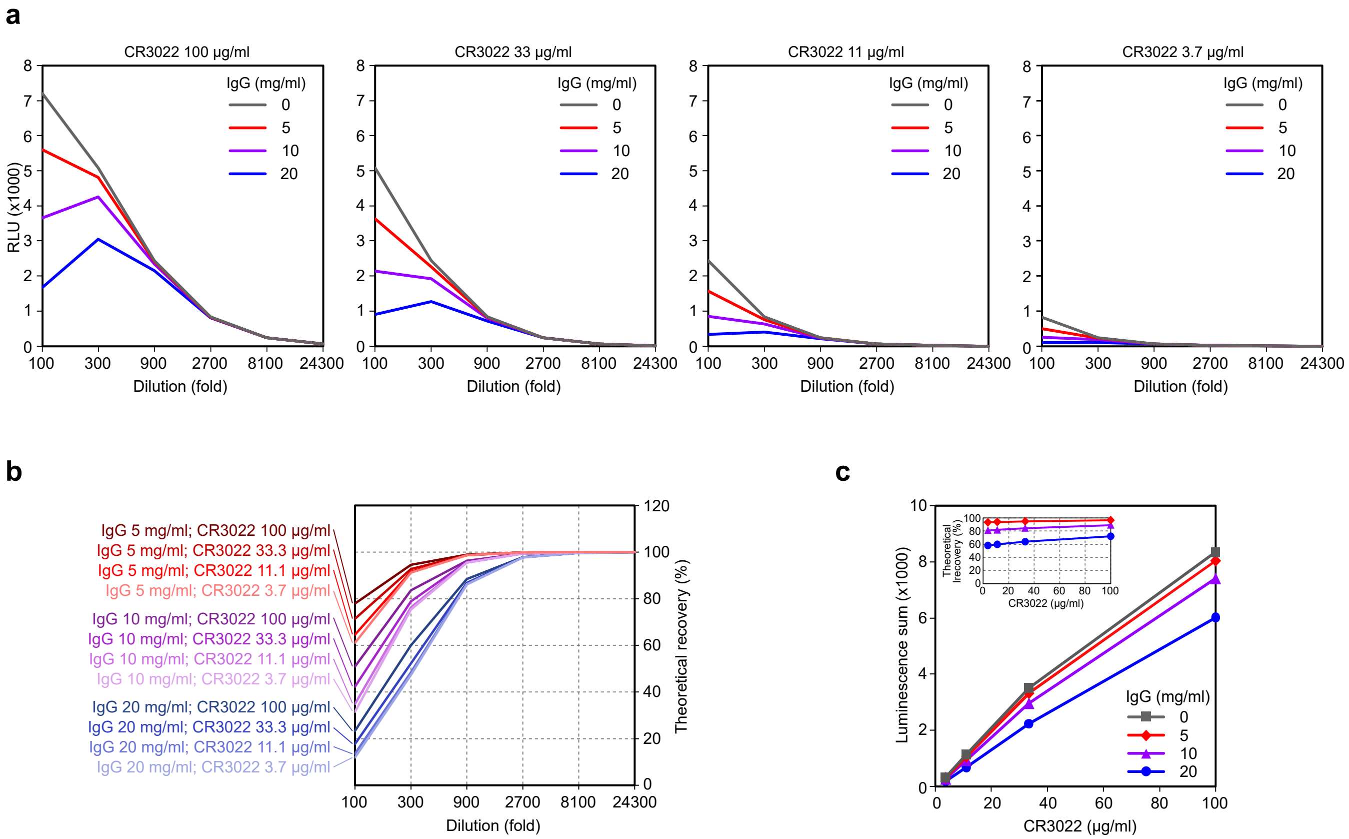

Supplementary Figure 2. Computational simulation of inhibitory effects of IgG on the SATiN assay. **A**. Virtual samples containing CR3022 (3.7, 11, 33 and 100 µg/mL) in the presence of IgG at different levels (5, 10, and 20 mg/mL) were serially diluted as indicated. Their tNLuc signals at each dilution endpoint were calculated using the chemical kinetics model derived from the experimental data shown in **Fig. 2B**. **B**. Theoretical recovery rates of samples in **A** at different dilutions were calculated by dividing the calculated reading of a virtual sample in **A** by the corresponding virtual sample not containing IgG. **C**. The overall tNLuc signal of each virtual sample was calculated by luminescence summation of the calculated signals at the 1:300, 1:900 and 1:2700 dilutions obtained in **A**. Overall recovery rates are presented inset.

Supplementary Figure 3

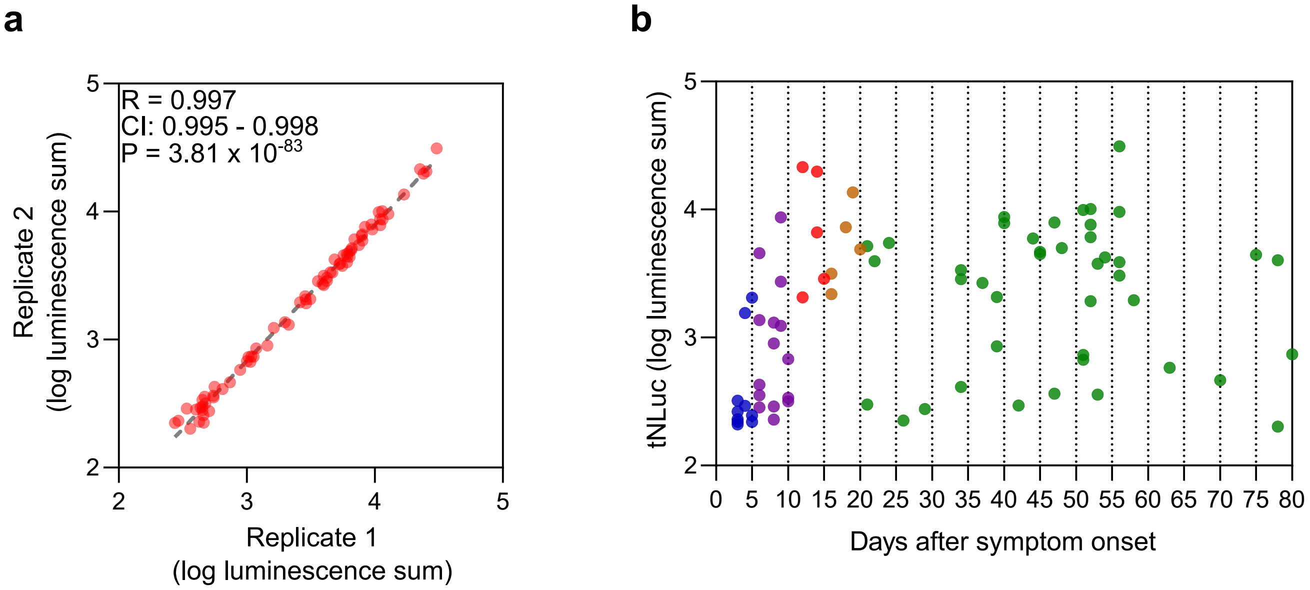

Supplementary Figure 3. Detection of α-SARS CoV-2 antibody in serum samples with the SATiN assay. **A**. 70 serum samples collected from COVID-19 patients or convalescents were analyzed in two independent replicates using the SATiN assay. The two sets of data are presented as a scatter plot. R and P values were obtained from two tailed Pearson analysis. B. SATiN luminescence signal of 80 samples plotted against the time of sample collection.
