# Supplementary material for: A homogeneous split-luciferase assay for rapid and sensitive detection of anti-SARS CoV-2 antibodies
Source: Nat Commun. 2021 Mar 22;12:1806. doi: 10.1038/s41467-021-22102-6 (PMC7985487; doi:10.1038/s41467-021-22102-6)
Supplement: Supplementary file 2 — Reporting Summary [file 41467_2021_22102_MOESM2_ESM.pdf]

nature research

Corresponding author(s): Shawn Owen
Igor Stagljar

Last updated by author(s): Feb 20, 2021

Reporting Summary

Nature Research wishes to improve the reproducibility of the work that we publish. This form provides structure for consistency and transparency in reporting. For further information on Nature Research policies, see our Editorial Policies and the Editorial Policy Checklist.

Statistics

For all statistical analyses, confirm that the following items are present in the figure legend, table legend, main text, or Methods section.

n/a	Confirmed	
☐	☒	The exact sample size (n) for each experimental group/condition, given as a discrete number and unit of measurement
☐	☒	A statement on whether measurements were taken from distinct samples or whether the same sample was measured repeatedly
☐	☒	The statistical test(s) used AND whether they are one- or two-sided<br>*Only common tests should be described solely by name; describe more complex techniques in the Methods section.*
☐	☒	A description of all covariates tested
☒	☐	A description of any assumptions or corrections, such as tests of normality and adjustment for multiple comparisons
☐	☒	A full description of the statistical parameters including central tendency (e.g. means) or other basic estimates (e.g. regression coefficient) AND variation (e.g. standard deviation) or associated estimates of uncertainty (e.g. confidence intervals)
☐	☒	For null hypothesis testing, the test statistic (e.g. F, t, r) with confidence intervals, effect sizes, degrees of freedom and P value noted<br>*Give P values as exact values whenever suitable.*
☒	☐	For Bayesian analysis, information on the choice of priors and Markov chain Monte Carlo settings
☒	☐	For hierarchical and complex designs, identification of the appropriate level for tests and full reporting of outcomes
☐	☒	Estimates of effect sizes (e.g. Cohen's d, Pearson's r), indicating how they were calculated

Our web collection on statistics for biologists contains articles on many of the points above.

Software and code

Policy information about availability of computer code

Data collection	BioTek Gen5 v3.09
Data analysis	Microsoft Excel 2016<br>GraphPad Prism 9

For manuscripts utilizing custom algorithms or software that are central to the research but not yet described in published literature, software must be made available to editors and reviewers. We strongly encourage code deposition in a community repository (e.g. GitHub). See the Nature Research guidelines for submitting code & software for further information.

Data

Policy information about availability of data

All manuscripts must include a data availability statement. This statement should provide the following information, where applicable:

- Accession codes, unique identifiers, or web links for publicly available datasets
- A list of figures that have associated raw data
- A description of any restrictions on data availability

All source data for the figures presented in the main manuscript and the Supplementary Information are included in the Source Data File. Relevant data are available upon request from the corresponding authors.

Field-specific reporting

Please select the one below that is the best fit for your research. If you are not sure, read the appropriate sections before making your selection.

[x] Life sciences [ ] Behavioural & social sciences [ ] Ecological, evolutionary & environmental sciences

For a reference copy of the document with all sections, see nature.com/documents/nr-reporting-summary-flat.pdf

Life sciences study design

All studies must disclose on these points even when the disclosure is negative.

Sample size	In Fig. 1-2, the sample sizes were determined by the capability of one test. In Fig. 3-4, the sizes were determined by the availability of the samples.
Data exclusions	All data are included.
Replication	Experiments in Fig. 1-2 were repeated 2-4 times. Experiments in Fig. 3-4 were repeated twice. All attempts at replication were successful.
Randomization	Randomization was not performed since the limitation of available human serum samples.
Blinding	Whenever necessary and/or possible, such as SATiN and neutralizing antibody assays, experiments were blindly conducted by different groups at different days.

Reporting for specific materials, systems and methods

We require information from authors about some types of materials, experimental systems and methods used in many studies. Here, indicate whether each material, system or method listed is relevant to your study. If you are not sure if a list item applies to your research, read the appropriate section before selecting a response.

Materials & experimental systems

n/a	Involved in the study	
[ ]	[x]	Antibodies
[ ]	[x]	Eukaryotic cell lines
[x]	[ ]	Palaeontology and archaeology
[x]	[ ]	Animals and other organisms
[ ]	[x]	Human research participants
[x]	[ ]	Clinical data
[x]	[ ]	Dual use research of concern

Methods

n/a	Involved in the study	
[x]	[ ]	ChIP-seq
[x]	[ ]	Flow cytometry
[x]	[ ]	MRI-based neuroimaging

Antibodies

Antibodies used	CR3022 is a published antibody with known reactivity to the RBD of SARS-CoV-1 and 2 and was produced in house.<br>α-human IgG polyclonal antibody conjugated with HRP: Jackson ImmunoResearch 109-035-098 (RRID: AB_2337586)
Validation	CR3022 was validated in this study and as previously reported (Abe, K. T. et al. A simple protein-based surrogate neutralization assay for SARS-CoV-2. JCI insight 5) which recognizes SARS-CoV-2 spike protein.<br>α-human IgG antibody conjugated with HRP was validated by the producer (https://www.jacksonimmuno.com/catalog/products/109-035-098). More references regarding α-human IgG can be found in database https://scicrunch.org/resources/Any/search?q=AB_2337586&l=AB_2337586

Eukaryotic cell lines

Policy information about cell lines

Cell line source(s)	HEK 293 cell line was kindly provided by Prof. Jason Moffat at University of Toronto and was originally purchased from ATCC.
Authentication	No authentication was performed.
Mycoplasma contamination	The cell line was not tested for mycoplasma.
Commonly misidentified lines (See ICLAC register)	No commonly misidentified cell lines were used.

Human research participants

Policy information about studies involving human research participants

Population characteristics	All samples were de-identified.
Recruitment	No participants were enrolled. All samples were preexisting.
Ethics oversight	All samples were enrolled through REB approved protocols, REB20-044c or REB 149-1994.

Note that full information on the approval of the study protocol must also be provided in the manuscript.
